# Parallel Evolution in Mosquito Vectors—A Duplicated Esterase Locus is Associated With Resistance to Pirimiphos-methyl in *Anopheles gambiae*

**DOI:** 10.1093/molbev/msae140

**Published:** 2024-07-10

**Authors:** Sanjay C Nagi, Eric R Lucas, Alexander Egyir-Yawson, John Essandoh, Samuel Dadzie, Joseph Chabi, Luc S Djogbénou, Adandé A Medjigbodo, Constant V Edi, Guillaume K Ketoh, Benjamin G Koudou, Faisal Ashraf, Chris S Clarkson, Alistair Miles, David Weetman, Martin J Donnelly

**Affiliations:** Department of Vector Biology, Liverpool School of Tropical Medicine, Liverpool, L3 5QA, UK; Department of Vector Biology, Liverpool School of Tropical Medicine, Liverpool, L3 5QA, UK; Department of Biomedical Sciences, University of Cape Coast, Cape Coast, Ghana; Department of Biomedical Sciences, University of Cape Coast, Cape Coast, Ghana; Department of Parasitology, Noguchi Memorial Institute for Medical Research, University of Ghana, Accra, Ghana; Department of Parasitology, Noguchi Memorial Institute for Medical Research, University of Ghana, Accra, Ghana; Laboratory of Infectious Vector Borne Diseases, Tropical Infectious Diseases Research Center (TIDRC), Université d’Abomey-Calavi (UAC), 01 B.P. 526 Cotonou, Benin; Laboratory of Infectious Vector Borne Diseases, Tropical Infectious Diseases Research Center (TIDRC), Université d’Abomey-Calavi (UAC), 01 B.P. 526 Cotonou, Benin; Research and Development Department, Centre Suisse de Recherches Scientifiques en Côte d’Ivoire, 01 BP 1303 Abidjan, Côte d’Ivoire; Department of Zoology, Faculty of Sciences, Laboratory of Ecology and Ecotoxicology, Université de Lomé, 01 B.P. 1515 Lomé, Togo; Research and Development Department, Centre Suisse de Recherches Scientifiques en Côte d’Ivoire, 01 BP 1303 Abidjan, Côte d’Ivoire; Department of Vector Biology, Liverpool School of Tropical Medicine, Liverpool, L3 5QA, UK; Wellcome Sanger Genomic Surveillance Unit, Wellcome Sanger Institute, Hinxton, Cambridge, CB10 1RQ, UK; Wellcome Sanger Genomic Surveillance Unit, Wellcome Sanger Institute, Hinxton, Cambridge, CB10 1RQ, UK; Department of Vector Biology, Liverpool School of Tropical Medicine, Liverpool, L3 5QA, UK; Department of Vector Biology, Liverpool School of Tropical Medicine, Liverpool, L3 5QA, UK; Wellcome Sanger Genomic Surveillance Unit, Wellcome Sanger Institute, Hinxton, Cambridge, CB10 1RQ, UK

**Keywords:** adaptation, insecticide resistance, anopheles, parallel evolution

## Abstract

The primary control methods for the African malaria mosquito, *Anopheles gambiae*, are based on insecticidal interventions. Emerging resistance to these compounds is therefore of major concern to malaria control programs. The organophosphate (OP), pirimiphos-methyl, is a relatively new chemical in the vector control armory but is now widely used in indoor-residual spray campaigns. While generally effective, phenotypic resistance has developed in some areas in malaria vectors. Here, we used a population genomic approach to identify novel mechanisms of resistance to pirimiphos-methyl in *A. gambiae s.l* mosquitoes. In multiple populations, we found large and repeated signals of selection at a locus containing a cluster of detoxification enzymes, some of whose orthologs are known to confer resistance to OPs in *Culex pipiens*. Close examination revealed a pair of alpha-esterases, *Coeae1f* and *Coeae2f*, and a complex and diverse pattern of haplotypes under selection in *A. gambiae, A. coluzzii* and *A. arabiensis*. As in *C. pipiens*, copy number variants have arisen at this locus. We used diplotype clustering to examine whether these signals arise from parallel evolution or adaptive introgression. Using whole-genome sequenced phenotyped samples, we found that in West Africa, a copy number variant in *A. gambiae* is associated with resistance to pirimiphos-methyl. Overall, we demonstrate a striking example of contemporary parallel evolution which has important implications for malaria control programs.

## Introduction

The spread of organophosphate (OP) resistance in the common house mosquito, *Culex pipiens*, is a textbook example of contemporary evolution in response to anthropogenic pressures (Raymond et al. 1998). In this species, mutations around two alpha-esterases enhanced the ability of the mosquito to detoxify OP insecticides used in larviciding campaigns (Gullemaud et al. 1997). This locus was termed *Ester*, with independent gene duplications and transposable element insertions at the *Est2* and *Est3* genes resulting in at least 16 distinct haplotypes across the mosquitoes” worldwide range (Raymond et al. 1996).

In the major malaria vector, *Anopheles gambiae*, resistance to OPs has historically been associated primarily with the *Ace1* locus, the target of OP and carbamate insecticides. At this locus, a complex combination of heterogeneous or homogeneous gene duplications and the *Ace1*-G119S non-synonymous mutation confer varying levels of resistance and fitness costs (Edi et al. 2014; Assogba et al. 2018; Grau-Bové et al. 2021). In recent years, OPs have been used increasingly for malaria vector control, primarily in indoor-residual spraying (IRS) campaigns as the formulation Actellic-300CS, in which pirimiphos-methyl (PM) is the active ingredient (Oxborough 2016). Resistance to the compound has been slow to arise, and the *Ace1* locus remains the only validated resistance marker (Grau-Bové et al. 2021). Importantly, resistance to compounds in indoor-residual spray formulations has been previously associated with IRS failure (Epstein et al. 2022).

In *An. gambiae*, genomics has facilitated the detection of genes involved in the insecticide resistance phenotype (Clarkson et al. 2018). Early transcriptomic studies implicated the involvement of metabolic and cuticular resistance mechanisms, first with microarray technology, and later, with RNA-Sequencing (David et al. 2005; Ingham et al. 2018; Nagi et al. 2023). More recently, whole-genome sequencing combined with population genetics has revealed the full extent of positive selection acting upon the wild genomes of *Anopheles* populations (Miles et al. 2017; Clarkson et al. 2020). These data have revealed a complex genetic architecture of insecticide resistance, including the involvement of amino acid mutations, copy number variants (CNVs), and cis-regulatory variation (Lucas et al. 2019; Clarkson et al. 2021; Dyer et al. 2023; Lucas et al. 2023; Kengne-Ouafo et al. 2024).

Given the recent widespread increase in the use of OPs in malaria vector control, we investigate whether there is evidence for the evolution of novel OP resistance mechanisms in *A. gambiae.* Using data from the *A. gambiae* 1,000 genomes project (Miles et al. 2017; Clarkson et al. 2020), we found evidence of large, repeated signals of selection at the locus orthologous to the *C. pipiens Ester* locus. This locus contains two alpha-esterases, *Coeae1f* and *Coeae2f*. We integrate expression data from studies across sub-Saharan Africa and perform an extensive analysis of this genomic region, which reveals that the *Coeae1f* and *Coeae2f* genes are an important resistance-associated locus. Two CNVs have arisen at this locus in *A. gambiae* and *A. arabiensis* and putative adaptive haplotypes have introgressed between *A. gambiae* and *A. coluzzii.* Lastly, we demonstrate that a CNV in *A. gambiae* is associated with resistance to the OP pirimiphos-methyl. These data illustrate the importance of parallel evolution and introgression in the evolution of adaptively important traits in insect disease vectors.

## Results

### Data Overview

**Fig. 1.**
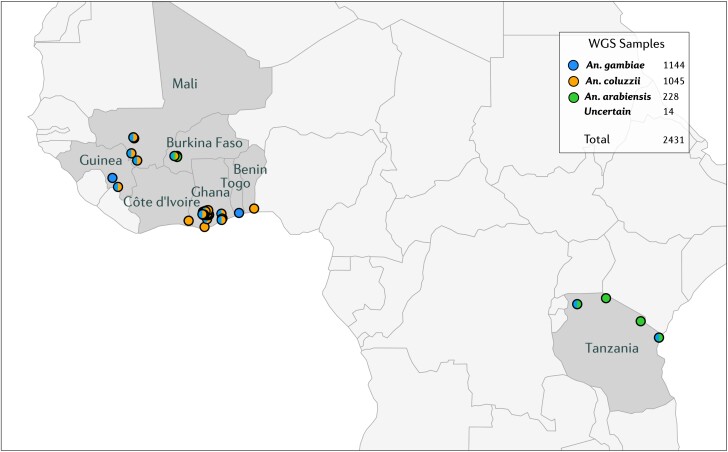
Map of sample sites for whole-genome sequenced individuals included in this study. Countries with collection sites are highlighted in gray and collection sites in circles. Collection sites are colored by the species which were sampled at that location. The sample inventory includes only samples which passed quality control.

We examined whole-genome sequence data from 2,431 individual mosquitoes collected across eight countries of sub-Saharan Africa between the years 2012 and 2017 (Fig. 1; supplementary table S1, Supplementary Material online). These data are public through the MalariaGEN Vector Observatory (Clarkson et al. 2020) and Genomics for African *Anopheles* Resistance Diagnostics (GAARD) projects (Lucas et al. 2023; see methods for further details on sample collections and sequencing). A subset of 828 of these mosquitoes were phenotyped against the OP insecticide pirimiphos-methyl (*n* = 347) or the type II pyrethroid, deltamethrin (*n* = 481). To ensure strong differentiation between phenotypic groups, mosquitoes were classified into whether they were alive after exposure to a high dose of insecticide (resistant) or dead after exposure to a low dose (susceptible). Full details of the bioassay protocol are given in the study by Lucas et al. 2023. For genome-wide selection scans and allele frequency estimates, we split the *A. gambiae* and *A. coluzzii* cohorts into early (2012 to 2015) and late (2017 to 2018) cohorts, to provide greater resolution to detect any low-frequency resistance mutants.

### A Novel Insecticide Resistance Locus

We investigated genome-wide signals of recent selection using the H12 statistic (Garud et al. 2015) in early (2012 to 2015) and late (2017 to 2018) cohorts of *A. gambiae*, *A. coluzzii*, and *An. arabiensis*, across all chromosomal arms. The H12 statistic is a measure of haplotype homozygosity; whereas haplotype homozygosity is calculated as the sum of squared haplotype frequencies, H12 combines the frequencies of the first and second most common haplotypes into a single frequency before taking the sum of squares. This allows H12 to be capable of detecting both hard and soft selective sweeps.

**Fig. 2.**
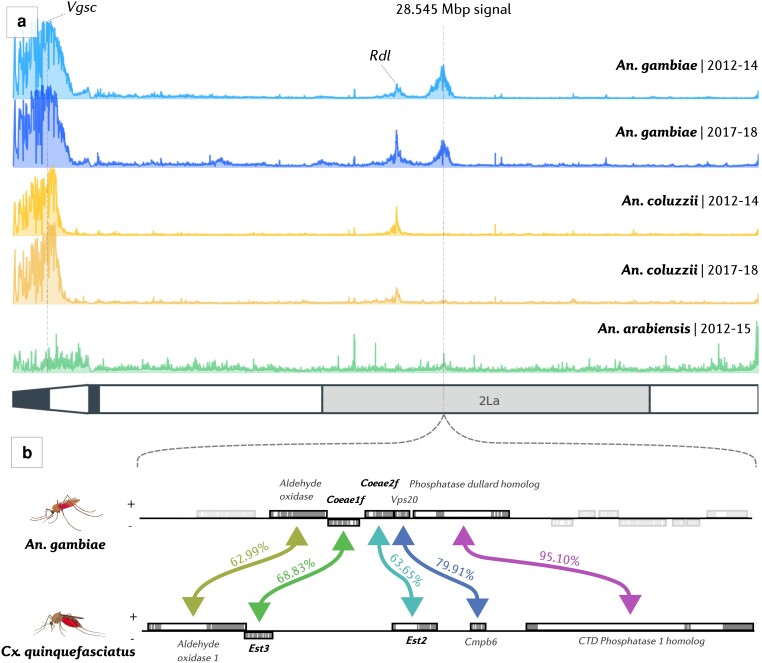
Selection scans and orthology of the alpha esterase cluster. a) H12 genome-wide selection scans on the 2L chromosomal arm, in early and later cohorts of *A. gambiae* (blue), *A. coluzzii* (orange), and *A. arabiensis* (green). H12 was applied in 1500 bp windows to phased, biallelic haplotype data. A diagram of the 2L arm is shown, with heterochromatin colored in black and the 2La inversion shaded. The location of the *Coeae1f/2f* locus (28.545 Mbp) is highlighted with a dashed gray line at 28.545 Mbp, and the known resistance loci *Vgsc* and *Rdl* are labeled. b) Orthology of the *An. gambiae* (AgamP4, 2L:28,530,000 to 28,580,000) *Coeae1f/2f* locus with the *C. quinquefasciatus Est2/Est3* locus (JHB2020, CM027412.1:137,360,000 to 137,410,000). One-to-one orthologous pairs are highlighted with colored arrows. Genes with orthologs not present within the 50 Kb window are grayed out. BLASTp protein sequence similarity is labeled for each orthologous pair.

In addition to known selection signals at the *Vgsc* and *Rdl* resistance-associated loci, a novel signal of selection was observed on the 2L chromosomal arm in multiple populations, at (≅28.545 Mb, Fig. 2a). This signal(s) was common in *A. gambiae* from West Africa and *A. arabiensis* from East Africa, but absent from *A. coluzzii*. The signals of selection are broad, with haplotype homozygosity inferred from the H12 signal extending beyond 1 Mb, suggesting that selection at the locus may have occurred relatively recently.

A closer examination of the region reveals several genes including two alpha-esterases, *Coeae1f* (AGAP006227) and *Coeae2f* (AGAP006228), that lie directly under the peak of the majority of the selection signals (Fig. 2). These alpha-esterases sit on opposing strands in reverse orientation, 495 bases apart, and contain a differing number of exons (*Coeae1f: 7*; *Coeae2f: 4*). At the amino acid level, sequence similarity is 50.93% between the primary transcripts of the two genes. To identify relationships with known insecticide resistance genes in other species, we performed reciprocal blast searches with two other major vectors, *Culex quinquefasciatus* (a member of the *C. pipiens* complex) and *Aedes aegypti* (supplementary table S2, Supplementary Material online). This revealed that the two carboxylesterases are one-to-one orthologs with the *Est3 (Coeae1f)* and *Est2 (Coeae2f)* carboxylesterases of *C. pipiens*, providing tentative evidence that *Coeae1f/2f* may be driving the signals of selection in *A. gambiae s.l.*, given the confirmed role of *Est2 and Est3* in OP resistance (Mouchès et al. 1986). The one-to-one orthologs in *A. aegypti* are AAEL010389 (*Coeae1f)* and AAEL017071 (*Coeae2f*), and although upregulated in some resistant strains (Strode et al. 2008), do not have a confirmed role in insecticide resistance. Figure 2b displays a 50 kb window in both the *A. gambiae* AgamP4 and *C. quinquefasciatus* JHB2020 genome assemblies, highlighting the orthology and synteny between the two species in this genomic region. *Coeae1f* shares 68.83% amino acid similarity with *Est3*, and *Coeae2f* shares 63.65% amino acid similarity with *Est2*. Despite other detoxification genes in the genomic region, we focus our analyzes on the two alpha-esterases due to the strength of evidence in their favor.

**Fig. 3.**
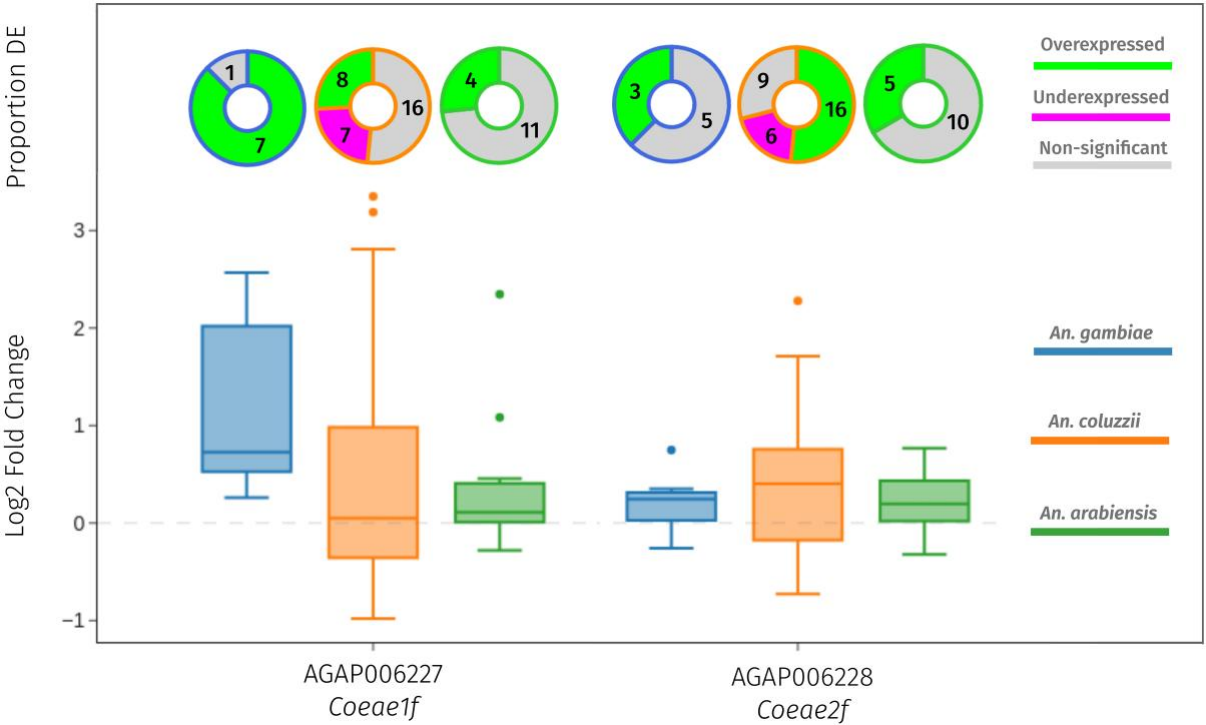
Gene expression data from AnoExpress. Data from a meta-analysis of 54 transcriptomic studies into insecticide resistance in *A. gambiae* (blue), *A. coluzzii* (orange), and *A. arabiensis* (green). Bottom) A box plot of log2-fold changes in 54 transcriptomic experiments from across sub-Saharan Africa. Top) Donut charts showing the proportion of experiments in each specific species which showed over-expression (green), under-expression (purple), or no DE (gray). The threshold for significance is an adjusted *P*-value of 0.05.

Insecticide resistance is often associated with increased expression of insecticide-detoxifying genes, known as metabolic resistance (Ingham et al. 2018). Given that the two carboxylesterases are detoxification enzymes and that the locus has been associated with increased gene expression in *C. pipiens*, we postulated that they may also exhibit elevated expression in resistant strains of *A. gambiae s.l.* To determine whether there was any evidence for resistance-associated differential expression (DE) in *Coeae1f* and *Coeae2f*, we used the tool *AnoExpress*, which integrates gene expression data from 31 microarray and 23 RNA-Sequencing experiments (Ingham et al. 2018; Nagi et al. 2023). Each of these experiments compared an insecticide-resistant strain of *A. gambiae s.l* to an insecticide-susceptible strain. Figure 3 displays log2-fold changes from each transcriptomic experiment, colored by species.

*Coeae1f* shows positive mean-fold changes in *A. gambiae* (2.25), *A. coluzzii* (1.45), *and A. arabiensis* (1.26), across all microarray and RNA-Sequencing studies. It is very commonly over-expressed in *A. gambiae* (7/8, 87.5% experiments), but less so in *A. coluzzii* (8/31, 25.8%) and *A. arabiensis* (4/15, 26.7%). *Coeae2f* also shows higher expression in resistant, compared to susceptible strains, with mean-fold changes of 1.15 in *A. gambiae*, 1.32 in *A. coluzzii*, and 1.16 in *A. arabiensis* (supplementary table S3, Supplementary Material online). Normalized read counts are high for both genes in the meta-analysis dataset (supplementary table S3, Supplementary Material online), suggesting that they are highly expressed at a base level, and so could still contribute to the insecticide-resistant phenotype without large fold changes. Overall, the expression data is suggestive of a potential role in insecticide resistance, however, there is little spatial or temporal overlap between the regions in which we observe large selection signals and the transcriptomic data itself. In the recent West African (WA) *A. gambiae* experiment (from Côte d’Ivoire), we observe a large fold change of 5.93 for *Coeae1f,* from a region with established pirimiphos-methyl resistance, though it cannot be confirmed that this population contains haplotypes under selection in our data.

### Diplotype Clustering of the *coeae1f/2f* Locus

To further explore patterns of selection at the *Coeae1f/2f* locus, we performed hierarchical clustering on diplotypes. A diplotype is a stretch of diploid genotypes, sometimes referred to as a multi-locus genotype. Diplotype clustering can help us to understand the nature of selection at a locus; for example, determining how many selective sweeps are ongoing, how many individuals harbor those sweeps, and whether there is evidence for haplotype sharing between cohorts. Typically, haplotype clustering is used for this purpose, however, we analyze unphased diplotypes rather than haplotypes to allow us to also resolve multiallelic amino acid mutations and CNVs, which are both typically challenging to phase onto haplotype scaffolds.

**Fig. 4.**
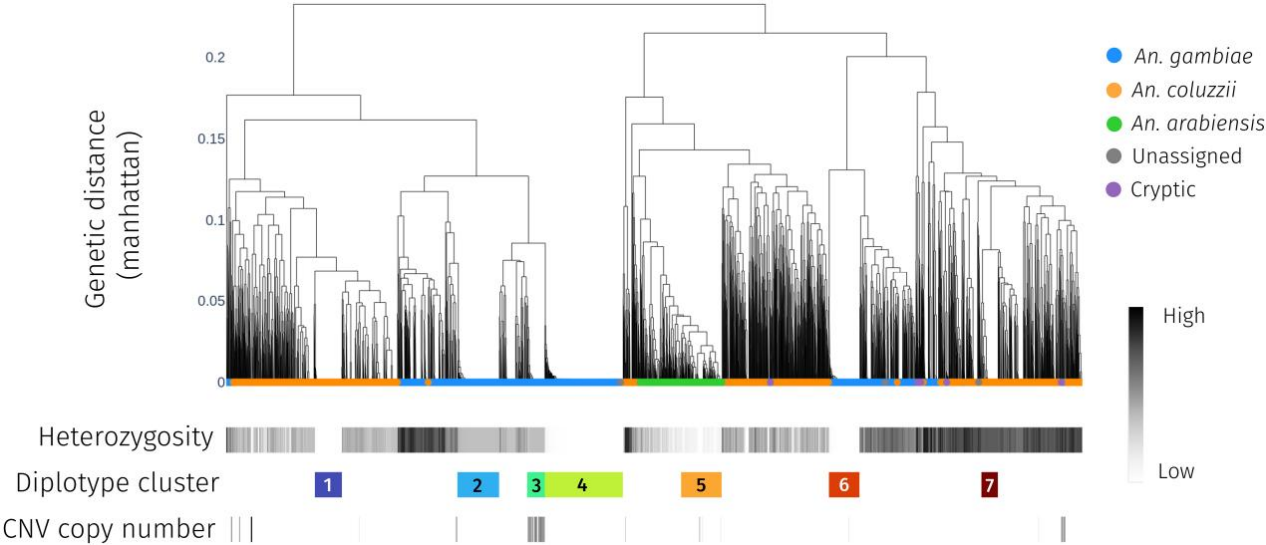
Diplotype clustering over the entire *Coeaexf* locus. We calculate pairwise distance between diplotypes spanning the start of Coeae1f (2L:28,545,396) to the end of Coeae2f (2L:28,550,748). Each column in the figure is a diplotype ordered by the dendrogram by hierarchical clustering, using genetic distance based on city-block (Manhattan) distance and complete linkage. The leaves of the dendrogram are colored by the species of the individual to which they belong. Note that due to overlapping points, not all dendrogram leaves can be seen. Underneath the dendrogram, the heterozygosity, assigned diplotype cluster, and CNV copy number of an individual are displayed as horizontal bars. Heterozygosity; individual-level heterozygosity was calculated as an average over all SNPs in the *Coeaexf* locus. Clusters with low sample heterozygosity or inter-sample genetic distances of 0 are indicative of a selective sweep. Diplotype clusters; diplotype clusters have been obtained by cutting the dendrogram at Manhattan distance of 0.04 with a minimum cluster size of 40 individuals. CNV copy number; CNV copy number of *Coeae1f* is shown as inferred by the HMM applied to normalized coverage data. A CNV is strongly associated with a sweep found in WA *A. gambiae (*cluster 3), and another one weakly associated with a sweep in Tanzanian *A. arabiensis* (cluster 5).

Figure 4 shows the results of this clustering, aligned with sample taxon, observed heterozygosity, the diplotype cluster a sample is assigned to, and the number of extra copies of *Coeae1f*. supplementary fig. S3, Supplementary Material online contains the same plot for *Coeae2f.* Selective sweeps can be identified where there are multiple diplotypes (the leaves of the dendrogram) with zero or very low genetic distances between each other, and are highlighted by numbered diplotype clusters. To obtain clusters of closely related diplotype clusters, we cut the tree at a genetic distance of 0.04 and set a minimum cluster size of 40. supplementary table S4, Supplementary Material online contains a summary table of diplotype clusters and associated metadata. In diplotype cluster 1, which is primarily *A. coluzzii* individuals, we also find two *A. gambiae*, suggestive of potential adaptive introgression between species. There is no other evidence of adaptive introgression in other diplotype clusters.

We investigated amino acid variation at the *Coeae1f* and *Coeae2f* genes by plotting amino acid variation alongside the diplotype clustering dendrograms (supplementary figs. S1 and S2, Supplementary Material online), to identify potential mutants driving selective sweeps. The majority of amino acid mutations found on selective sweep diplotypes are also found at moderate to high frequencies in wild-type individuals and are therefore not obvious candidates to be causal mutations. There are, however, some exceptions—*Coeae1f-*E477 V is relatively rare outside of swept diplotypes, as are *Coeae1f-*Q129E, *Coeae1f-*D463N, *Coeae2f-S*357N, and *Coeae2f-*V516L.

### Copy Number Variation

In *C. pipiens*, CNVs at the *ester* locus that cover one or both of the *Coeae1f* and *Coeae2f* orthologs have spread around the world (Raymond et al. 1991). Given the gene expression data and the presence of copy number variation at this locus in *C. pipiens*, we speculated whether CNVs might exist at these loci in *A. gambiae s.l*. There is increasing evidence of CNVs and metabolic resistance associations (Lucas et al. 2019, 2023; Njoroge et al. 2022). To identify CNVs, we calculated the copy number at each gene in the region and then computed the frequency of amplifications or deletions in the dataset.

The combined copy number and diplotype clustering analysis suggested that CNVs were associated with two selective sweeps, one strongly associated with diplotype cluster 3, found in *An. gambiae*, and one very weakly with diplotype cluster 5, found in *A. arabiensis* (Fig. 4). In the *A. gambiae* diplotype cluster that is associated with a CNV (cluster 3), heterozygosity is elevated, suggesting that this cluster contains individuals heterozygous for the CNV sweep haplotype and a distinct, separate haplotype under selection. We found the *A. gambiae* CNV only in WA populations of *A. gambiae*, and we called this duplication *Coeaexf*-Dup1. The CNV in *A. arabiensis* was found in a cohort from Tanzania, we call this *Coeaexf*-Dup2. supplementary fig. S3, Supplementary Material online shows the copy number of *Coeae1f* for each diplotype cluster.

**Fig. 5.**
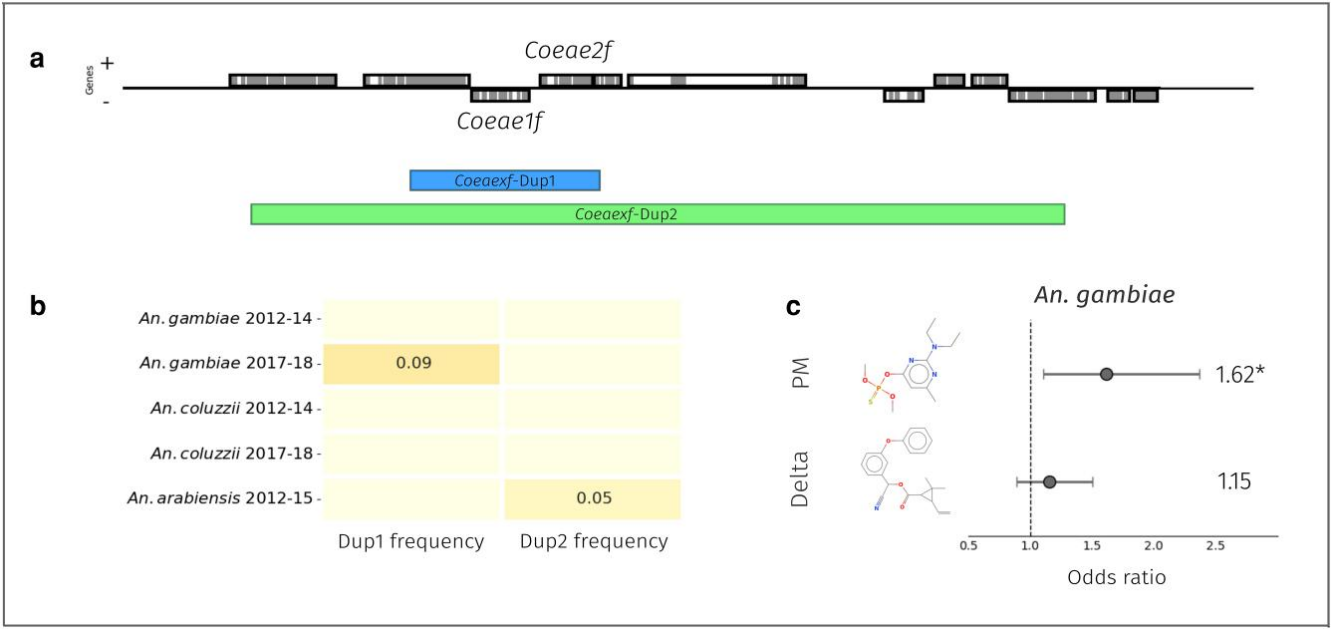
A schematic of the *Coeae1f/2f* locus, with associated frequency and genotype:phenotype associations. a) A schematic of the locus from the *A. gambiae* PEST reference genome. Exons are colored in gray and introns in white. The genomic spans of *Coeaexf-*Dup1 and *Coeaexf-*Dup2 are displayed below the gene track, colored by the species in which they are found (blue: *A. gambiae*, green: *A. arabiensis*). b) Frequencies of the presence of at least one Dup allele in our cohorts. c) A forest plot showing odds ratios from the binomial GLM assessing the relationship between *Coeae1f* CNV copy number and survival to either pirimiphos-methyl or deltamethrin, when controlling for CNVs at *Ace-1* the other major OP-resistance locus.

We then examined the genomic span of each copy number variant. supplementary fig. S4, Supplementary Material online shows sequence coverage data in 300 bp windows for two representative individuals from Ghana and Tanzania. Both CNVs cover the two carboxylesterases *Coeae1f* and *Coeae2f*, and the *Coeaexf*-Dup1 CNV in Ghana and Togo only covers these two genes—also amplifying a truncated version of a putative aldehyde oxidase, AGAP006226 (Fig. 5a). *Coeaexf*-Dup2 is much larger and spans ten genes in total. Figure 5b shows a summary of the frequencies of CNVs at the locus in each cohort. In our data, we find that individuals positive for *Coeaexf-*Dup1 have a maximum copy number of six, whereas those positive for *Coeaexf-*Dup2 have a maximum copy number of four. No other CNVs were noted covering detoxification genes at the locus.

### A CNV at *Coeae1f/Coeae2f* is Associated With Resistance to Pirimiphos-methyl in WA *An. Gambiae*

We hypothesized that copy number variation at *Coeae1f/2f* may confer resistance to OP insecticides used in vector control. We specifically tested for associations against the active ingredient of Actellic-300CS, pirimiphos-methyl, and pyrethroid deltamethrin. Deltamethrin is the most widely used insecticide in long-lasting insecticide-treated nets (LLINs), and due to the ubiquitous use of pyrethroids, resistance has spread through sub-Saharan Africa over the past two decades.

To determine whether CNVs in *Coeae1f* and *Coeae2f* are associated with resistance to insecticides, we took a subset of samples from our *A. gambiae* cohorts which were phenotyped by World Health Organisation (WHO) tube assays to either pirimiphos-methyl or deltamethrin. Details of this sample set have been published previously (Lucas et al. 2023). Reliable CNV data were available from 463 *A. gambiae* samples from four locations. We found CNVs in *Coeae1f* in 28 out of 146 samples (19%) from Baguida (Togo) and 23 out of 160 (14%) samples from Obuasi (Ghana), but such CNVs were absent from all 37 samples in Aboisso (Côte d'Ivoire) and 120 samples in Madina (Ghana). We tested for a significant association between an individual's CNV copy number (the inferred number of copies of *Coeae1f* and *Coeae2f*) and phenotype, in both Baguida and Obuasi. We also included the CNV copy number in *Ace1* in the model due to its known association with pirimiphos-methyl resistance. When considering each population separately, we found a significant association with PM resistance in Obuasi (*P* = 0.034, odds ratio [OR] = 1.76, 1.04 to 3.4) and marginally non-significant in Baguida (*P* = 0.077). When combining the two locations and including location as a random effect in the model, significance was increased compared to either site alone (*P* = 0.007, OR = 1.62, 1.1 to 2.36). In contrast, as expected, we found no significant association of *Coeae1f* CNVs with resistance to deltamethrin.

### A CNV Diagnostic Assay

To track these CNVs in time and space, we have developed PCR primers for use in standard genotyping PCRs, which involve three different primers for each duplication. A forward and reverse primer combines to produce a band when the duplication is present. There is then also a control primer, which combines with either the forward or reverse primer to produce a control band, which should be present in all samples regardless of genotype. Primer sequences can be found in supplementary Text S1, Supplementary Material online. We tested these primers on a set of 20 samples which were either positive or negative for *Coeaexf-*Dup1 from West Africa, or positive or negative for *Coeaexf-*Dup2 from Tanzania. Concordance was 100% (supplementary fig. S5, Supplementary Material online), though we note that very faint “duplication” bands can be seen for the *Coeaexf-*Dup1 negative samples, and so these samples should only be called positive if there is a similar level of intensity to the control band.

## Discussion

In the common house mosquito, *C. pipiens*, the *ester* locus, orthologous to the *Coeae1f/2f* locus, is a textbook example of contemporary anthropogenic selection (Raymond et al. 1998). In an adaptive response to exposure to toxic OP insecticides, at least 16 haplotypes spread throughout the range of *C. pipiens*, competing with each other and providing varying combinations of insecticide resistance and fitness costs. Many of these haplotypes were associated with gene amplifications around the two alpha-esterases *Est2* and *Est3*.

In this study, we used whole-genome sequence data from 2,431 individual mosquitoes to demonstrate parallel evolution at the orthologous locus in malaria mosquitoes. There is evidence of positive selection acting upon the locus in *A. gambiae*, and *An. arabiensis*, with multiple diplotypes rising to moderate frequencies, some of which harbor CNVs. In part due to the extreme genetic diversity of *Anopheles gambiae s.l*, resistance loci are often characterized by harboring multiple selective sweeps with separate evolutionary origins (Miles et al. 2017).

We also demonstrate that *Coeae1f* and to a lesser extent *Coeae2f*, are commonly over-expressed when comparing insecticide-resistant to insecticide-susceptible cohorts in sub-Saharan Africa. The upregulation of detoxification enzymes is a common evolutionary strategy found in insects to protect against xenobiotic exposure (Liu 2015). The *Coeae1f* and *Coeae2f* transcripts are also present at a high base level even in susceptible mosquito strains, implying that moderate fold-change over-expression could have an outsized phenotypic impact, when compared to naturally lowly expressed genes. Due to inherent limitations of microarray technology that make absolute quantification challenging, early transcriptomic studies relied on relative fold-changes alone, and so findings may have been biased toward lowly expressed genes such as *Cyp6p3* and *Cyp6 m2,* which typically display high relative fold-changes (Kwiatkowska et al. 2013; Nagi and Ingham 2024). Later, evidence from copy number variation and population genomics implicated *Cyp6aa1* and *Cyp9k1*, genes with high base-level expression but lower relative fold changes (Njoroge et al. 2022; Djoko Tagne et al. 2024; Nagi and Ingham 2024).

We demonstrate that in *A. gambiae* a CNV at this locus is associated with resistance to the OP pirimiphos-methyl. We expect that in this context, the CNVs identified will function to increase the expression of genes which they encompass, as has been shown at other resistance loci in *A. gambiae* (Njoroge et al. 2022). We cannot rule out, however, that other variants linked to the CNV are playing a role in this resistance. Some similarities between species are striking—in *C. pipiens* there is a gene amplification allele which, as with the *Coeaexf*-Dup1 allele in *A. gambiae*, covers both alpha-esterases and a partial copy of the neighboring aldehyde oxidase gene (Buss *a*nd Callaghan 2004). Five of the seven diplotype clusters reported in this study do not contain CNVs, and it is not clear what causal variants are driving these selective sweeps; amino acid mutations are a possibility, however, genomic insertions, which are commonly involved in insecticide resistance, could also be important, as well as variation in *cis*-regulatory elements (Rostant et al. 2012; Hu et al. 2021).

As well as evidence of parallel evolution, the study also provides important information for malaria control programs. We reveal a novel locus in *A. gambiae* which contributes to resistance to the OP pirimiphos-methyl. Pirimiphos-methyl, formulated as Actellic CS300, is widely used in IRS campaigns throughout sub-Saharan Africa. Before this study, only one locus in *A. gambiae s.l* had been associated with resistance to this compound—*Ace1*, which is the target site of OP and carbamate insecticides. Unlike LLINs which still provide a physical barrier and so protect even against insecticide-resistant vectors, IRS is arguably more prone to resistance-mediated failure. Examples of this come from Kwazulu-Natal, where pyrethroid-resistant *Anopheles funestus* caused a malaria outbreak after a change in IRS product from DDT to pyrethroids (Maharaj et al. 2005) and more recently, from IRS campaigns in Uganda (Epstein et al. 2022). It is therefore even more important to detect emerging mechanisms of resistance early, so that insecticide resistance management (IRM) practices can be employed, such as rotating out the insecticide in favor of the new IRS active ingredients such as clothianidin, broflanilide, and chlorfenapyr. The discoveries of mutations at both the *Ace1* and *Coeaexf* loci in *An. gambiae* mosquitoes from West Africa are important, and these mutations should be monitored as part of IRM campaigns. We provide a CNV diagnostic PCR assay allowing researchers to monitor these variants in time and space. We stress that the discovery of this novel variant does not necessarily mean pirimiphos-methyl is no longer effective, but that this locus should be incorporated in future surveillance activities.

## Materials and Methods

### Data Collection and Whole-genome Sequencing

We used a subset of whole-genome sequence variation data from phase 3 of the *Anopheles* 1,000 genomes project (Ag 1,000 g) and a recent genome-wide association study (GWAS) study in West Africa (Clarkson et al. 2020; Lucas et al. 2023). The data contained 2,431 individual *Anopheles* mosquitoes, of which there were 1,144 *A. gambiae*, 1,045 *A. coluzzii*, 228 *A. arabiensis*, 11 of a cryptic taxon, and 3 unassigned individuals. Specimens were all collected between 2013 and 2017. Sample provenance and methods for genome sequencing and analysis are described elsewhere (Clarkson et al. 2020; Lucas et al. 2023). In brief, Illumina short-read data were aligned to the AgamP4 reference genome using BWA version 0.7.15, and single nucleotide polymorphisms (SNPs) called with GATK UnifiedGenotyper version 3.7.0. Phasing of biallelic variants was performed with a combination of read-backed phasing (WhatsHap version 1.0) and statistical phasing with SHAPEIT4 version 4.2.1. Data are stored on Google Cloud and are directly accessible via the malariagen_data python package.

### Genome-wide Selection Scans

We first performed genome-wide selection scans with the H12 statistic (Garud et al. 2015). This statistic is a measure of haplotype homozygosity, and captures is particularly powerful in detecting soft selective sweeps, or where there are multiple distinct haplotypes under selection at the same locus. We first extracted phased biallelic haplotypes from the 2,431 individual mosquitoes and split the populations into cohorts of WA *A. coluzzii*, WA *A*. *gambiae*, East African (EA) *A. gambiae* and EA *A. arabiensis*. We further split the WA cohorts into early (2012 to 2014) and later collections (2017 to 2018). We then took a random sample of 100 haplotypes from each cohort and used malariagen_data to compute H12 in 1,500 SNP stepping windows.

### Orthology Searches

We extracted the predicted amino acid sequences of AGAP006227-RA and AGAP006228-RA from Vectorbase. Both genes have only a single transcript annotated in the PEST reference (AgamP4.12). We used diamond v2.1.8 (Buchfink et al. 2015) to perform sequence alignment (BLASTP) with the *A. gambiae* PEST, *C. quinquefasciatus* JHB2020, and *A. aegypti* LVP_AGWG protein reference genomes (Vectorbase, version 66), reporting the top five best hits. We then perform reciprocal searches by performing sequence alignment of the candidate *C. quinquefasciatus* orthologs (CQUJHB000812 and CQUJHB006176) back to the *A. gambiae* PEST reference to confirm one-to-one orthology.

### Gene Expression Data

We used the Python package *AnoExpress* (Nagi et al. 2023) to load and visualize gene expression data for *Coeae1f* and *Coeae2f*. *AnoExpress* contains gene expression data from microarray (*n* = 31) and RNA-sequencing (*n* = 23) studies into insecticide resistance in *A. gambiae s.l* and *A. funestus.* We extracted data from *A. gambiae*, *A. Coluzzii*, or *A. arabiensis*, and calculated the mean and median fold-change and read counts for each species. We used the adjusted *P*-values from the *AnoExpress* DESeq2 analysis to calculate the number of significant up or downregulated experiments (*P*_adj_ <= 0.05). Fold-change data were averaged over both RNA-Sequencing and microarray studies, whereas read count data were derived only from the RNA-Sequencing studies.

### Diplotype Clustering

We extracted diplotypes from the start of *Coeae1f* to the end of the *Coeae2f* gene (2L:28,545,396 to 28,550,748) and clustered the diplotypes with complete-linkage hierarchical clustering. Diplotypes, sometimes referred to as multi-locus genotypes, are stretches of diploid genotypes. We first transform diplotypes into an array of allele counts, that is, for each site, an array of size four, one for each possible allele. For each site, we then find the pairwise difference between this allele counts array for each individual in the pair. We then use city-block (Manhattan) distance as the distance metric and complete linkage. We also determined which non-synonymous variants were present on each diplotype and plotted this data alongside sample heterozygosity over the region, and the number of extra copies of *Coeae1f* (AGAP006227) and *Coeae2f* (AGAP006228).

### Sample Heterozygosity

We included heterozygosity in the diplotype clustering plots, to permit easy identification of diplotype clusters homozygous for a selective sweep, as opposed to diplotype clusters which contain two separate haplotypes under selection. To calculate individual-level heterozygosity, we used the scikit-allel v1.3.7 implementation (allel.heterozygosity_observed). For a given diplotype region, we calculated the observed heterozygosity for each variant and then averaged these per-variant heterozygosity values to get a single value of heterozygosity for the region for each individual sample.

### CNV Detection and CNV Association Tests

To detect novel CNV alleles at the Coeaexf locus, we used the Ag1000 g coverage-based CNV calls, which applies a hidden Markov model (HMM) to normalized sequencing coverage to estimate the copy number state in 300 bp genomic windows as previously described (Lucas et al. 2019). In brief; sequencing coverage is first normalized to account for variation in local nucleotide composition. A HMM is then applied to the normalized, filtered, coverage data. The HMM contains 13 hidden states, representing copy numbers from 0 to 12 in increments of 1, allowing the detection of up to a 6-fold amplification of a genetic region, or 10 extra copies.

Using copy number estimated for 828 phenotyped individuals from West Africa (Lucas et al. 2023) we performed CNV association tests. These individuals were phenotyped against either Pirimiphos-methyl or Deltamethrin, using WHO tube bioassays (WHO, 2016). We excluded samples with a high variance in coverage above 0.2, leaving 463 samples with reliable CNV calls. High variance in coverage can lead to erratic CNV calls. We first estimated the copy number at *Coeae1f.* We performed CNV association tests for each location with a binomial generalised linear model (GLM) with logit link function using CNV copy number and *Ace1* genotype as predictor variables and phenotype (dead or alive) as the response variable. GLMs were performed in R v4 (R Core Team 2018) and the inclusion of location as a random effect was implemented using the package glmmTMB (Brooks et al. 2017).

## Supplementary Material

msae140_Supplementary_Data

## Data Availability

Codes used to analyze the data are available in the GitHub repository https://github.com/sanjaynagi/coeaexf. All sequencing, alignment, SNP, and CNV calling were carried out as part of the Anopheles gambiae 1000 genomes project v3.2 (https://www.malariagen.net/data).

## Supplementary Material

Supplementary  material is available at *Molecular Biology and Evolution* online.
