# Supplementary material for: Parallel Evolution in Mosquito Vectors—A Duplicated Esterase Locus is Associated With Resistance to Pirimiphos-methyl in Anopheles gambiae
Source: Mol Biol Evol. 2024 Jul 10;41(7):msae140. doi: 10.1093/molbev/msae140 (PMC11267716; doi:10.1093/molbev/msae140)
Supplement: msae140_Supplementary_Data [file msae140_supplementary_data.pdf]

***Coeaexf* supplementary information**

Supplementary Table 1. Sample manifest

country	year	arabiensis	coluzzii	gambiae	cryptic	unassigned
**Benin**	2017	0	90	0	0	0
**Burkina Faso**	2012	0	82	99	0	0
**Burkina Faso**	2014	3	53	46	0	0
**Cote d'Ivoire**	2017	0	1	36	0	1
**Ghana**	2012	0	64	36	0	0
**Ghana**	2017	0	0	398	0	0
**Ghana**	2018	0	690	63	0	0
**Guinea**	2012	0	11	125	0	0
**Mali**	2012	0	27	65	0	2
**Mali**	2014	0	27	33	0	0
**Tanzania**	2012	87	0	0	0	0
**Tanzania**	2013	1	0	32	10	0
**Tanzania**	2015	137	0	32	1	0
**Togo**	2017	0	0	179	0	0

Supplementary Table 2. Reciprocal best hits.

Aligning AGAP006227-PA, AGAP006228 protein sequences against the *Culex quinquefasciatus* JHB2020 and Aedes aegypti reference genomes. For the *Culex* genes, we then align back to the *An. gambiae* PEST reference. Only the top five hits for each search are shown.

CQUJHB000812 = *Est3* , CQUJHB006176 = *Est2*

query_seqid	reference_seqid	Protein identity %	length	mismatch	evalue
AGAP006228-RA	CQUJHB006176.P9455	64.2	542	190	1.76E-264
AGAP006228-RA	CQUJHB000812.P1285	51	537	257	6.7E-199
AGAP006228-RA	CQUJHB006077.P9314	36.1	529	309	3.05E-90
AGAP006228-RA	CQUJHB012326.P19058	34.6	552	329	3.31E-85
AGAP006228-RA	CQUJHB012326.P19057	34.6	552	329	4.27E-85
AGAP006227-RA	CQUJHB000812.P1285	68.8	539	165	1.97E-292
AGAP006227-RA	CQUJHB006176.P9455	50.7	534	258	4.74E-197
AGAP006227-RA	CQUJHB006077.P9314	35	545	322	8.31E-91
AGAP006227-RA	CQUJHB005542.P8540	34.1	557	321	3.61E-89
AGAP006227-RA	CQUJHB012326.P19058	33.8	541	331	3.94E-88
CQUJHB000812	AGAP006227-PA	68.8	539	165	3.59E-291
CQUJHB000812	AGAP006228-PA	51	537	257	2.42E-202
CQUJHB000812	AGAP002391-PA	35.5	552	324	8.14E-93
CQUJHB000812	AGAP006700-PA	32.7	556	345	1.53E-84
CQUJHB000812	AGAP006727-PA	34	550	330	1.11E-83
CQUJHB006176	AGAP006228-PA	64.2	542	190	1.78E-267
CQUJHB006176	AGAP006227-PA	50.7	534	258	2.42E-195
CQUJHB006176	AGAP002391-PA	38.9	458	259	7.33E-84
CQUJHB006176	AGAP006700-PA	33.1	553	343	7.07E-81
CQUJHB006176	AGAP006726-PA	36.4	456	279	1.72E-79
AGAP006228-RA	AAEL017071-PA	64.4	534	189	3.29E-268
AGAP006228-RA	AAEL010389-PA	48.9	542	273	1.89E-192
AGAP006228-RA	AAEL019679-PB	33.8	553	332	2.38E-83
AGAP006228-RA	AAEL019679-PC	33.8	553	332	2.38E-83
AGAP006228-RA	AAEL019679-PD	33.8	553	332	2.38E-83
AGAP006227-RA	AAEL010389-PA	66.8	539	176	1.13E-283
AGAP006227-RA	AAEL017071-PA	52.2	533	249	3.94E-207
AGAP006227-RA	AAEL019679-PB	35.9	541	320	7.61E-95
AGAP006227-RA	AAEL019679-PC	35.9	541	320	7.61E-95

AGAP006227-RA	AAEL019679-PD	35.9	541	320	7.61E-95

Supplementary Figure 1. Amino acid variation at Coeae1f

NB. diplotype cluster assignments and colours may differ to the diplotype clustering plot in the main text, as the main text used the entire Coeaexf locus, rather than this plot which focuses on *Coeae1f* alone.

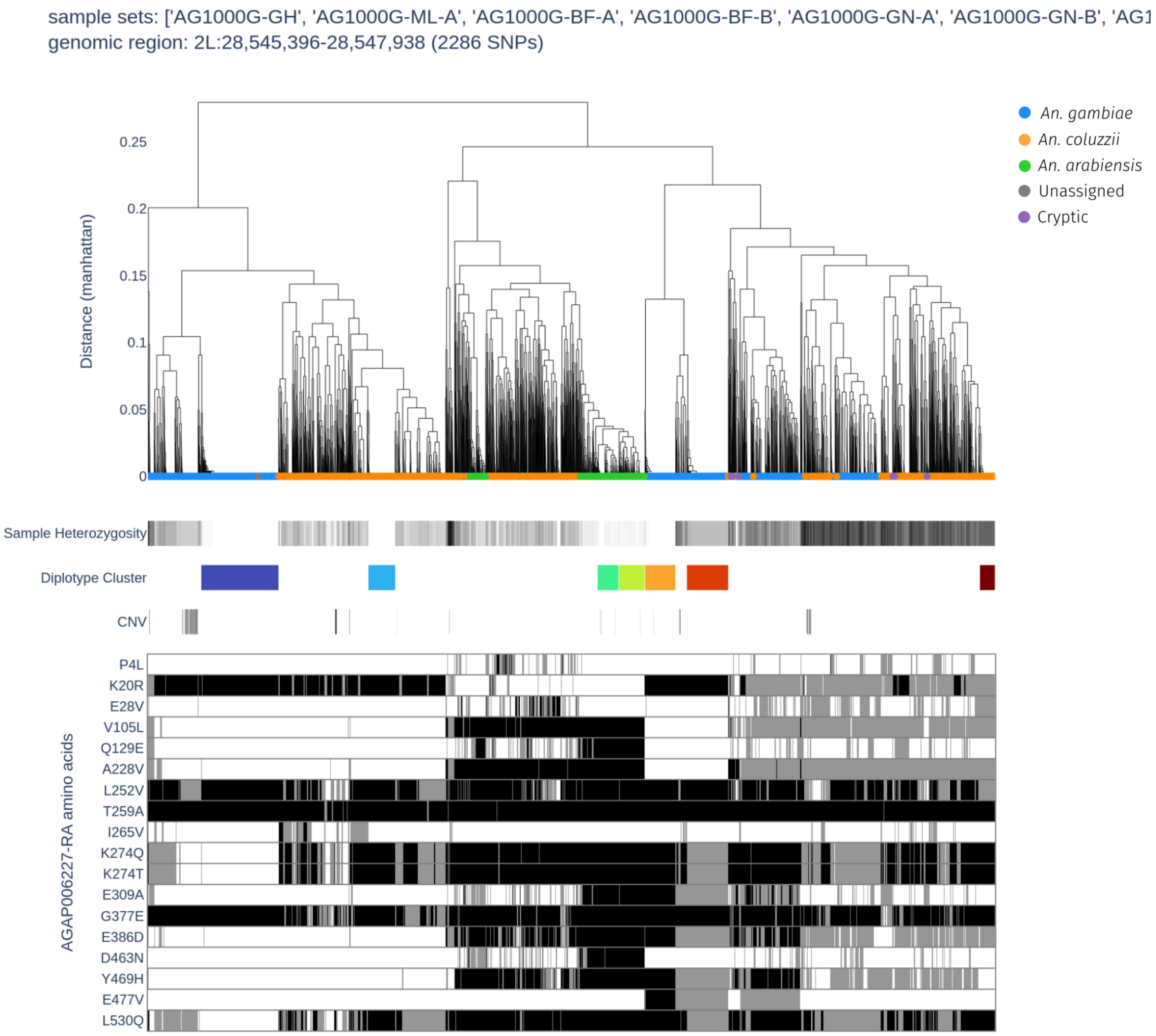

Supplementary Figure 1. **Diplotype clustering over the *Coeae1f* region.** We calculate pairwise distance between diplotypes spanning the start of *Coeae1f* to the end of *Coeae1f*. Each column in the figure is a diplotype ordered by the dendrogram by hierarchical clustering, using genetic distance based on city-block (Manhattan) distance and complete linkage. The leaves of the dendrogram are coloured by the species of the individual to which they belong. Note that due to overlapping points, not all dendrogram leaves can be seen. Underneath the dendrogram, the heterozygosity, assigned diplotype cluster, and CNV copy number of an individual are displayed as horizontal bars. Heterozygosity; individual-level heterozygosity was calculated as an average over all SNPs in the *Coeaexf* locus. Clusters with low sample heterozygosity or inter-sample genetic distances of zero are indicative of a selective sweep. Diplotype clusters; diplotype clusters have been obtained by cutting the dendrogram at manhattan distance of 0.04 with a minimum cluster size of 40 individuals. CNV copy numbers; CNV copy number of *Coeae1f* is shown as inferred by the HMM applied to normalised coverage data Amino acid variation is displayed below the dendrogram.

Supplementary Figure 2. Amino acid variation at Coeae2f

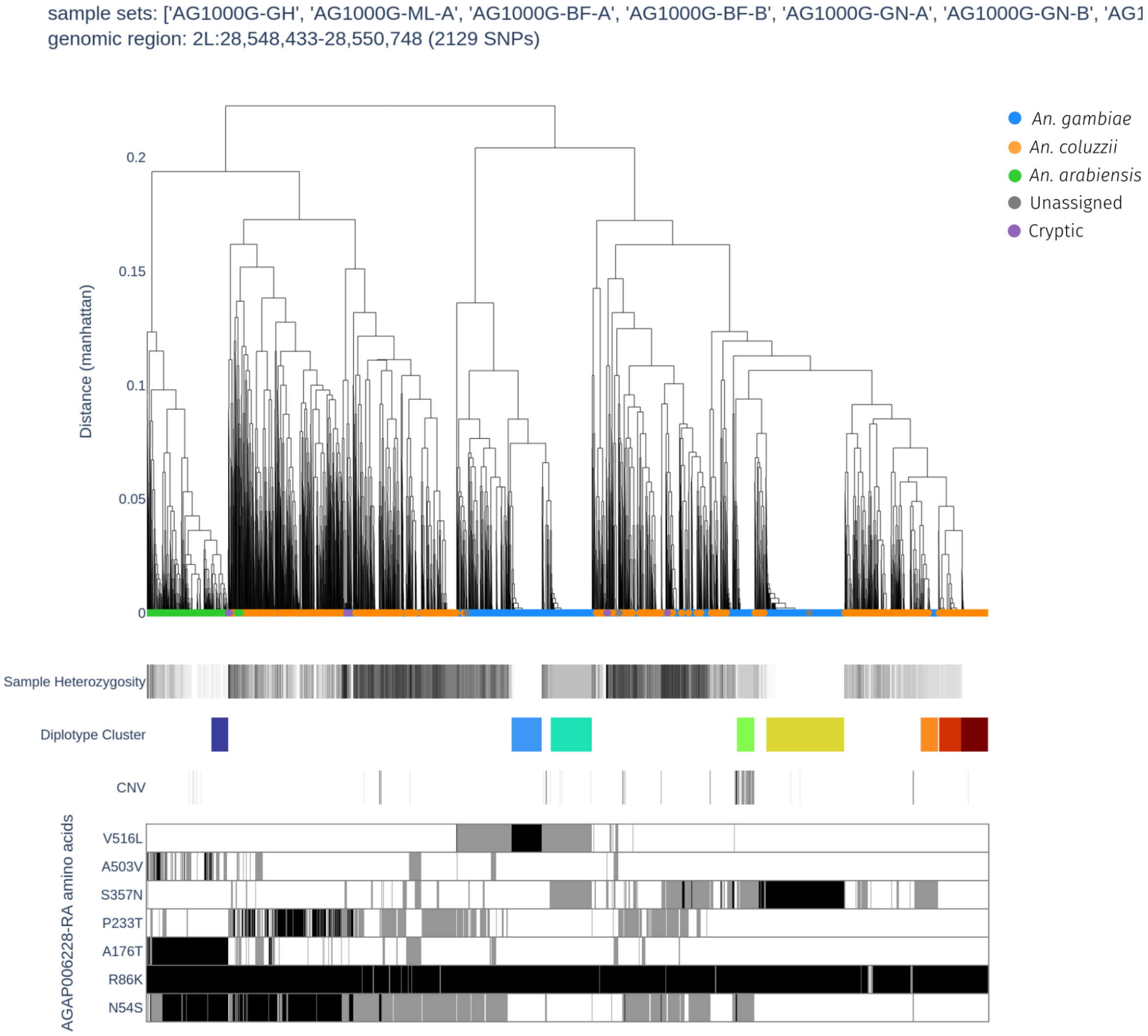

Supplementary Figure 2. **Diplotype clustering over the *Coeae2f* region.** We calculate pairwise distance between diplotypes spanning the start of *Coeae2f* to the end of *Coeae2f*. Each column in the figure is a diplotype ordered by the dendrogram by hierarchical clustering, using genetic distance based on city-block (Manhattan) distance and complete linkage. The leaves of the dendrogram are coloured by the species of the individual to which they belong. Note that due to overlapping points, not all dendrogram leaves can be seen. Underneath the dendrogram, the heterozygosity, assigned diplotype cluster, and CNV copy number of an individual are displayed as horizontal bars. Heterozygosity; individual-level heterozygosity was calculated as an average over all SNPs in the *Coeaexf* locus. Clusters with low sample heterozygosity or inter-sample genetic distances of zero are indicative of a selective sweep. Diplotype clusters; diplotype clusters have been obtained by cutting the dendrogram at manhattan distance of 0.04 with a minimum cluster size of 40 individuals. CNV copy numbers; CNV copy number of *Coeae1f* is shown as inferred by the HMM applied to normalised coverage data. Amino acid variation is displayed below the dendrogram.

Supplementary Table 3. A summary of expression data of Coeae1f/2f.

Species	GeneID	N up sig	N down sig	Total experiments	Median FC	Mean FC	Median normalised count	Mean normalised count
**arabiensis**	AGAP006227	4	0	15	1.08	1.26	373.51	334.71
**arabiensis**	AGAP006228	5	0	15	1.15	1.16	760.08	646.63
**coluzzii**	AGAP006227	8	7	31	1.04	1.45	372.22	432.61
**coluzzii**	AGAP006228	16	6	31	1.32	1.32	754.83	844.19
**gambiae**	AGAP006227	7	0	8	1.65	2.25	319.57	410.59
**gambiae**	AGAP006228	3	0	8	1.19	1.15	590.18	563.87

Supplementary Table 4. Diplotype cluster summary

This table describes the number of individuals in our data who fall into each diplotype cluster, and the proportion of that cluster with CNV-positive individuals.

cluster	arabiensis	coluzzii	gambiae	Proportion with cnv
**WT**	113	925	666	0.02
**1**	0	74	2	0
**2**	0	0	118	0.01
**3**	0	0	50	0.91
**4**	0	0	221	0
**5**	115	0	0	0.09
**6**	0	0	87	0.01
**7**	0	46	0	0

Supplementary Figure 3. Diplotype clusters and CNV Status/ Taxon

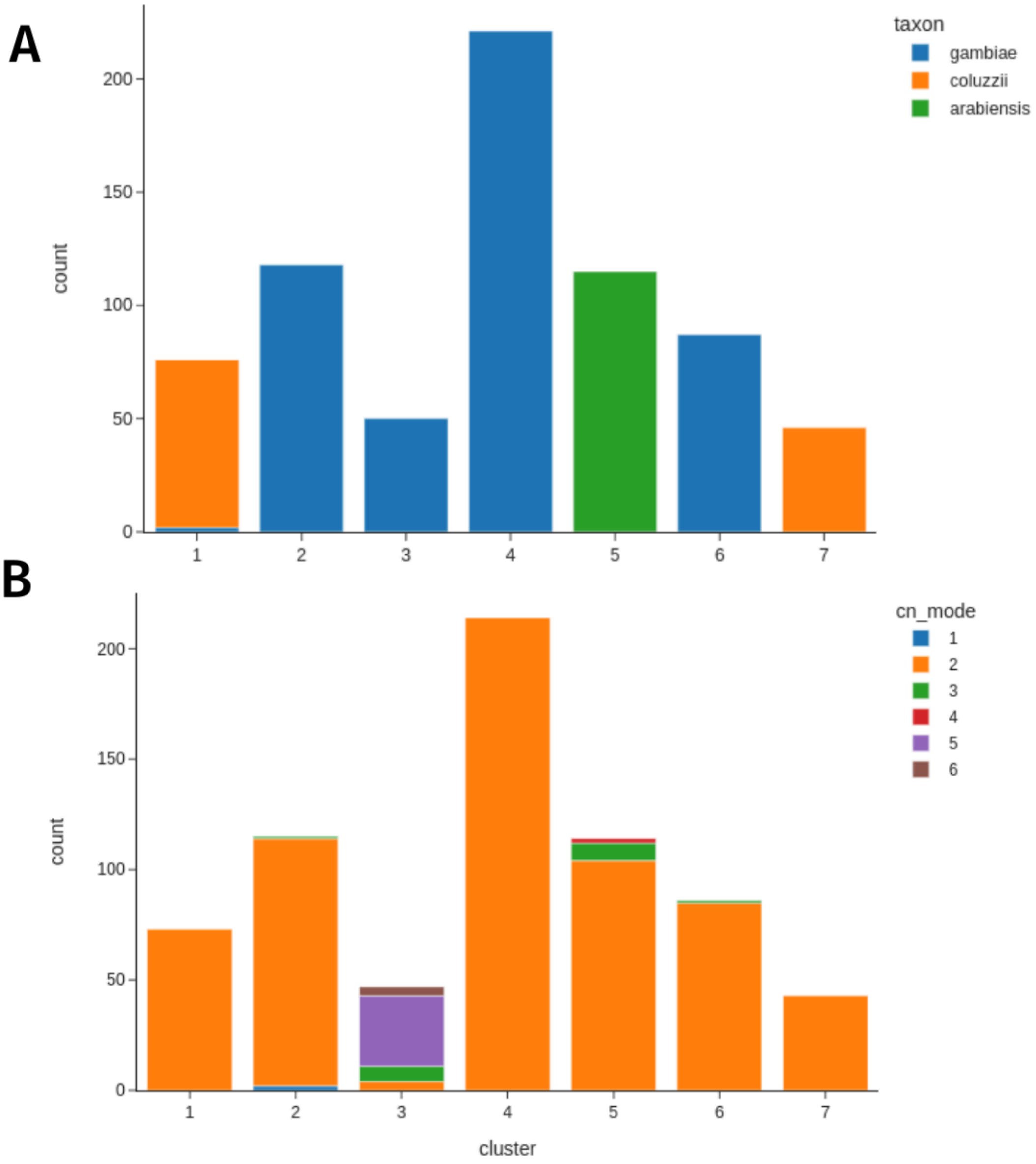

Supplementary Figure 3A) The number of individuals in each diplotype cluster, coloured by taxon assignment. 3B) The number of individuals in each diplotype cluster, coloured by copy number at Coeae1f.

Supplementary Figure 4. Example coverage traces for Dup1 and Dup2

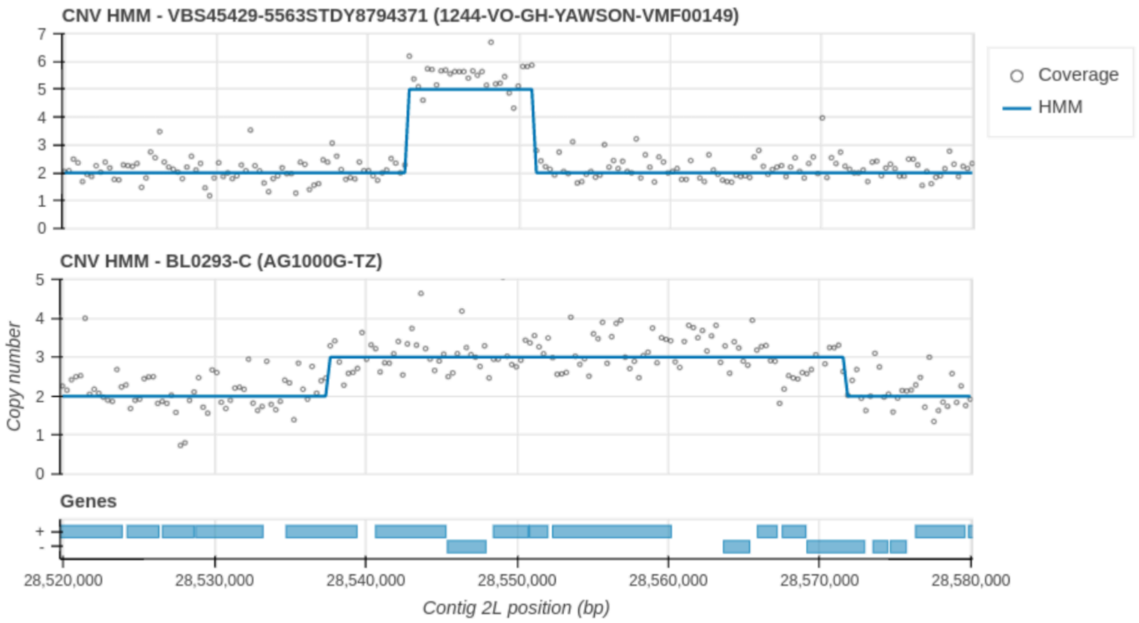

Suppl. Figure 4. Example coverage traces for Dup1 (upper, *An. gambiae female* collected in Obuasi, Ghana) and Dup2 (lower, *An. arabiensis female* from Moshi, Tanzania). Coverage is calculated in 300 Bp stepping windows, and the line represents the HMM prediction of copy number state described in (Lucas *et al.*, 2019).

Supplementary Text 1 - CNV primer diagnostic protocols

Primer name	Sequence
Coeaexf_Dup1_2F	TTTTGCGGTCCATGCACGAT
Coeaexf_Dup1_2R	GAGCCGTCGAAGATGTCCTT
Coeaexf_Dup1_2Rc1	GCTTTTCCAGCGTTTCCAGC
Coeaexf_Dup2_2F	AATGTACCCGTTCAGCAGCT
Coeaexf_Dup2_2R	CGGCAGATGTTACCACCGAA
Coeaexf_Dup2_2Rc1	TGTGCAGCACTATCTGGAGG

For all primer sets, cycling conditions are:

94°C	3mins
35 cycles of:	
94°C	30s
60°C	30s
72°C	45s
72°C	10mins

Coeaexf_Dup1_2 primers:
Expected CNV band size: 178
Expected control band size: 429
<u>per reaction:</u>

water	2.95ul
10x PCR buffer	1ul
2mM dNTPs	1ul
5mM primer Coeaexf_Dup1_2F	2ul
5mM primer Coeaexf_Dup1_2R	1ul
5mM primer Coeaexf_Dup1_2Rc1	1ul
Taq	0.05ul

DNA	1ul
Total	10ul

Coeaexf_Dup2_2 primers:
Expected CNV band size: 167
Expected control band size: 400
per reaction:

water	2.95ul
10x PCR buffer	1ul
2mM dNTPs	1ul
5mM primer Coeaexf_Dup2_2F	2ul
5mM primer Coeaexf_Dup2_2R	1ul
5mM primer Coeaexf_Dup2_2Rc1	1ul
Taq	0.05ul
DNA	1ul
Total	10ul

Supplementary Figure 5 - CNV primer PCR validation

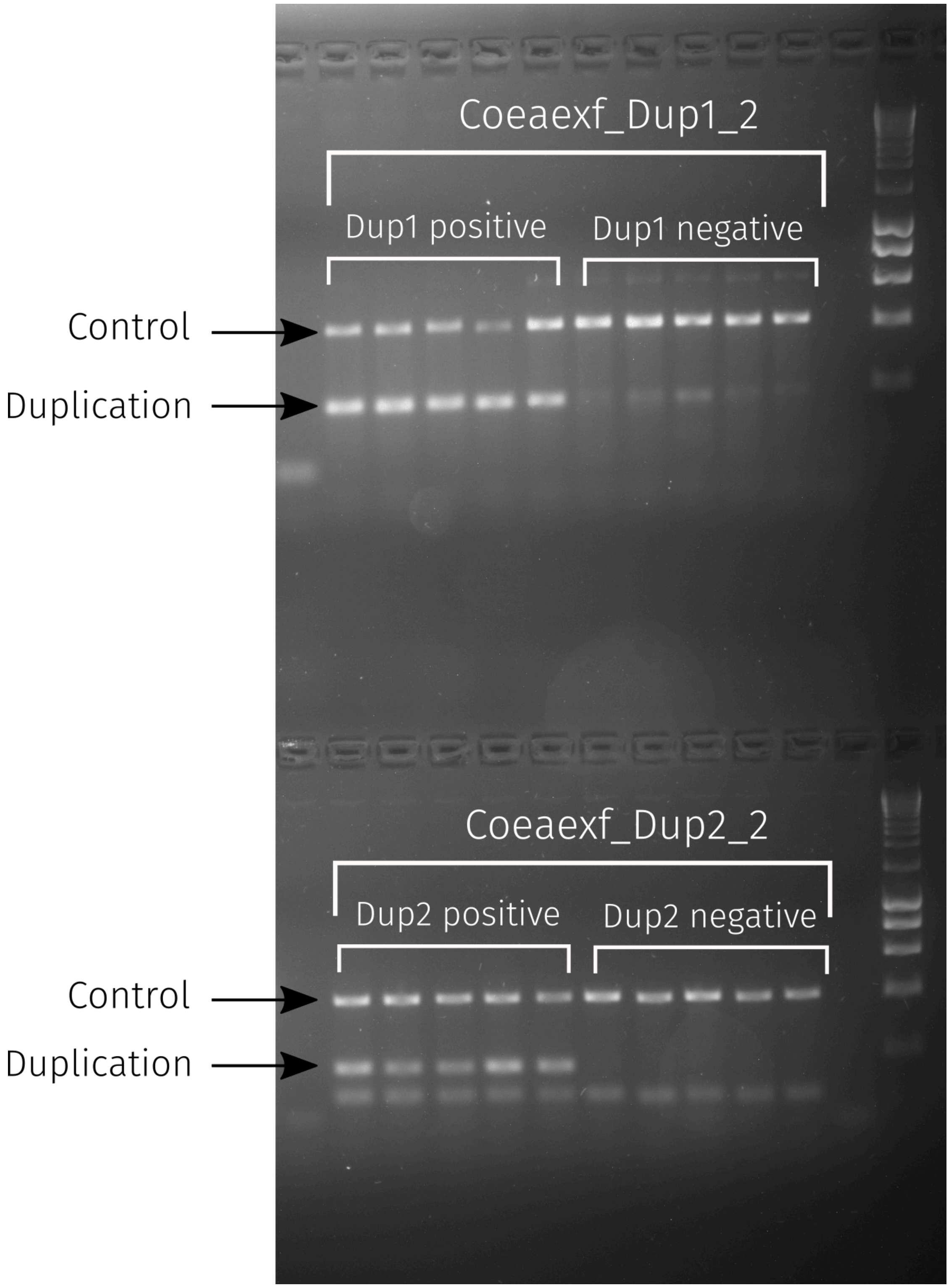

Supplementary Figure 5. A gel image of the two duplication diagnostic PCR primers, applied to *An. gambiae* (Dup1) and *An. arabiensis* (Dup2) individuals. Five Dup-negative samples and five Dup-positive samples were tested for each primer pair. Concordance is 100%.
